# Differences in patient-reported symptoms following radical gastrectomy: a propensity score-matched cohort study

**DOI:** 10.3389/fonc.2026.1574289

**Published:** 2026-03-25

**Authors:** Yuan Xie, Li Li, Rui Xu, Qiong Gu, Shuo-meng Xiao, Hong-Li Ma

**Affiliations:** Department of gastric surgery, Sichuan Cancer Hospital, Chengdu, China

**Keywords:** gastrectomy, gastric cancer, operative treatment, patient-reported symptoms, propensity score matching

## Abstract

**Background:**

Surgery is not only the mainstay of treatment for gastric cancer but also the key to improving postoperative quality of life for patients. Patient-reported symptoms following radical gastrectomy tend to be diverse and descriptive. This study aims to describe patient self-reported outcomes and compare the differences between total gastrectomy (TG), distal gastrectomy (DG), and proximal gastrectomy (PG).

**Methods:**

This was a retrospective comparative cohort study that included 578 patients. All these patients were diagnosed with gastric cancer and underwent gastrectomy. The included patients were divided into the TG group, DG group, and PG group. All these data were collected and registered prospectively during the patient’s postoperative follow-up process. The analyzed data in this study included food intake, weight loss, sleep disorders, and the most common digestive symptoms, at the postoperative first week (1W), the first postoperative month (1M), the third postoperative month (3M), and the sixth postoperative month after discharge (6M). These data were retrospectively compared in these three groups. Propensity score matching (PSM) was used to decrease selection bias. A 0.02 caliper width was used.

**Results:**

After PSM, TG, DG, and PG were compared with each other. Compared with DG and TG, food intake recovered more quickly in PG at 1W (*P* < 0.05). Food intake in most patients recovered to the normal diet at 3M. 46% of patients (263/578) experienced sleep disorders at 1W. No matter if it is TG, DG, or PG, easy to wake was the main complaint of patients with the insomnia symptom. Compared with TG and PG, weight loss was lowest in DG (*P* < 0.05). Incidence of digestive symptoms was lowest in DG (*P* < 0.05). In three groups (TG, DG, and PG), abdominal distention was the most reported discomfort. Compared with DG, the incidence of eating obstruction sensation was higher in TG (*P* = 0.04) and in PG (*P* = 0.01).

**Conclusions:**

Compared with DG and PG, weight loss was maximum, and the incidence of digestive symptoms was highest in TG. The study found that sleep disorders and abdominal distension were the most common complaints after gastrectomy. Some drugs should be given to relieve these symptoms, and further research is needed to improve the quality of life for these patients.

## Introduction

Gastric cancer (GC) is one of the common digestive malignant tumors worldwide, especially in East Asia (1). Despite there being an increasing trend in the incidence of early GC, the advanced-stage GC also accounts for the majority of cases. In the present day, surgery is still the mainstay of treatment. According to the size of the tumor, location of the tumor, and (2–4)clinical stage of the tumor, the extent of gastrectomy with the appropriate procedure is selected and includes total gastrectomy (TG), distal gastrectomy (DG), and proximal gastrectomy (PG). However, studies have shown that different surgical approaches leading to varying postoperative complications and different anastomotic methods associated with distinct digestive symptoms both significantly impact patients’ quality of life after surgery (5–7). Proximal gastrectomy (PG) is typically suitable for patients with malignant tumors in the proximal gastric body or cardia. The main complications following this procedure include reflux esophagitis and anastomotic stenosis (8, 9). Consequently, subsequent research has explored various reconstruction methods, such as double-tract reconstruction, esophagogastrostomy, gastric tube reconstruction, jejunal interposition, jejunal pouch interposition, and the double-flap technique, to alleviate digestive symptoms 5. Total gastrectomy (TG) is the primary method of radical treatment for locally advanced proximal gastric cancer (GC), as well as for most diffuse-type GC and other gastric tumors. Major postoperative complications include significant weight loss, vitamin B/D deficiency, and dumping syndrome (10, 11). Adverse emotional states such as depression and anxiety have also been observed (12). Therefore, reconstruction methods such as jejunal pouch interposition appear to mitigate these adverse outcomes (13). Distal gastrectomy (DG) is the standard surgical approach for distal gastric cancer. Bile reflux is a common postoperative complication that also carries an increased risk of residual gastric cancer (14–16). Billroth-I and Roux-en-Y are the two most common methods for gastrointestinal reconstruction. However, debate continues regarding which technique is superior (17). Billroth I is widely recommended due to its physiological and anatomical advantages as well as surgical simplicity (18). However, the hyperosmotic anastomosis and higher recurrence rate of gastric cancer associated with Billroth I make it unsuitable for every patient (19). Furthermore, numerous studies have indicated that compared with B-I reconstruction, R-Y reconstruction is associated with lower incidence rates of postoperative complications, including reduced anastomotic leakage, less bile reflux, and fewer cases of residual gastritis (14, 20, 21). Additionally, studies have also indicated that adverse reactions such as postoperative reflux and gastric stasis caused by Billroth-I or Roux-en-Y reconstruction are related to the size of the remnant stomach. When the remnant stomach is small (approximately one-quarter of the original stomach volume), Roux-en-Y reconstruction is more frequently recommended (22–24). These studies collectively highlight the growing emphasis on postoperative quality of life for gastric cancer patients. For patients eligible for surgical resection, although radical gastrectomy and digestive tract reconstruction techniques continue to evolve and improve, evidence regarding their efficacy in alleviating postgastrectomy syndromes and enhancing postoperative quality of life remains insufficient (22). Patient-reported outcomes (PROs ) serve as a more direct assessment indicator, as patient self-evaluation of postoperative quality of life can provide a more effective comparison of differences among various surgical procedures (25). Moreover, studies in other fields have demonstrated that such self-reported data can be utilized to formulate appropriate treatment plans, improve the quality of care, and evaluate treatment efficacy (26, 27). These results, as they represent patients’ subjective accounts, not only assist in formulating more suitable surgical plans during the perioperative period but also provide more precise information for personalized treatment in the future. Furthermore, and most importantly, patient self-reports serve as an excellent method for describing post-gastrectomy symptoms, as these accounts are subjective, authentic, and appropriately reflect the patient’s health status, the impact of surgery on daily life, and psychological well-being. To our knowledge, there is currently limited research on the types and differences of adverse postoperative symptoms caused by various gastrectomy procedures. Therefore, this study aims to analyze patient-reported outcomes to quantify and characterize the primary discomforts following gastrectomy and specifically compare symptom profiles across three surgical approaches: TG, DG, and PG.

## Methods

### Patients and study design

This was a retrospective comparative cohort study. Informed consent was obtained before collecting data. The study protocol was approved by the Ethics Committees of the Hospital and complied with the principles of the Declaration of Helsinki. The inclusion criteria were as follows: diagnosed with gastric cancer; radical gastrectomy was performed: TG, DG, or PG; and laparoscopy or open surgery was not limited. The exclusion criteria were as follows: follow-up data were incomplete; insomnia was diagnosed before surgery. Based on surgical methods, patients were divided into three groups: TG, DG, and PG. In our hospital, a double-tract anastomosis was performed for PG. So, all patients who were included in the PG group were performed with double-tract anastomosis in this study.

### Data collection

**Figure 1:**
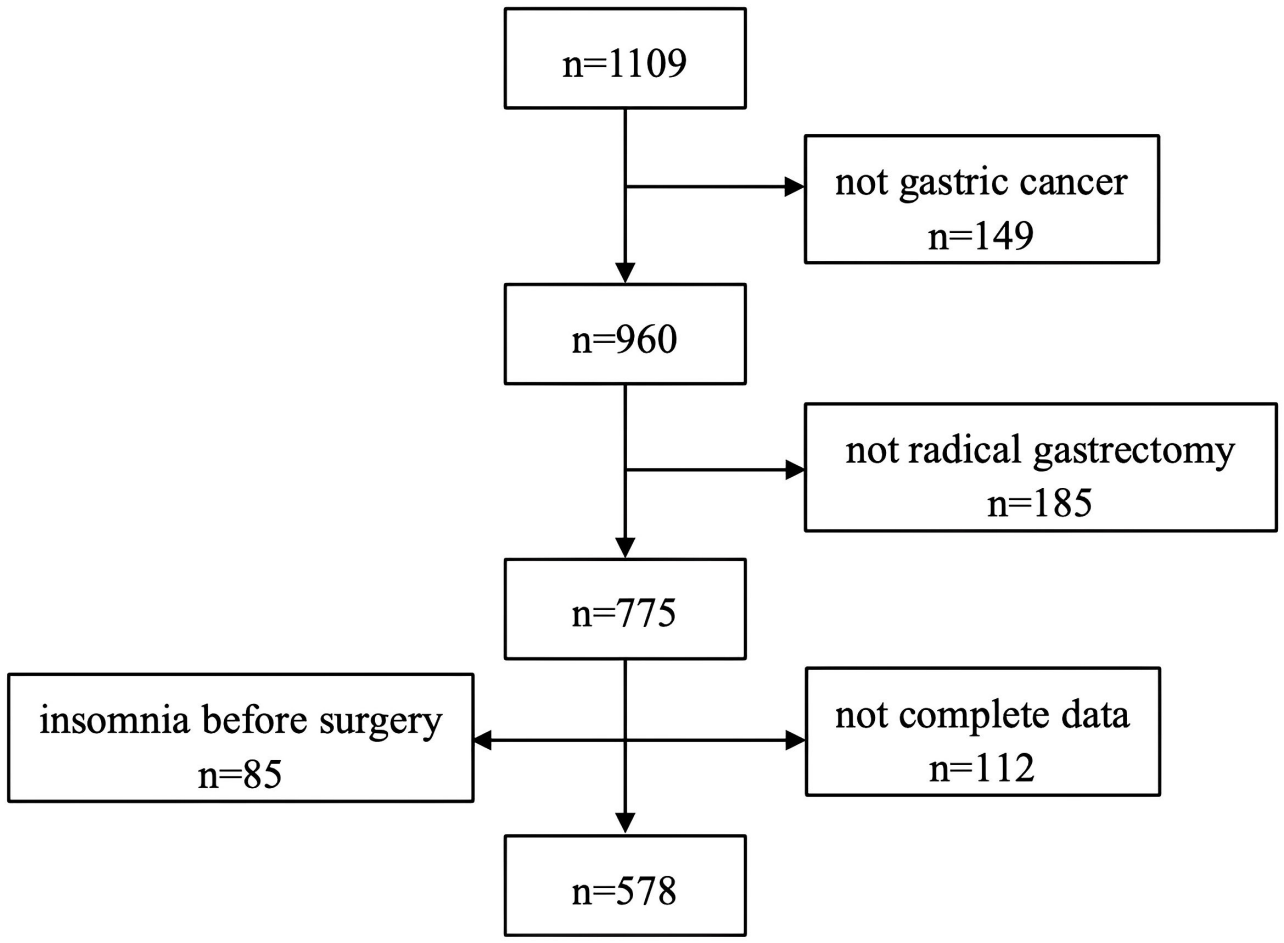
Flowchart of patient selection. Study participants were screened according to the inclusion and exclusion criteria, resulting in 578 eligible patients prior to propensity score matching (PSM).

All these data were collected prospectively during the patient’s postoperative follow-up process by our team. A questionnaire, including food intake, weight loss, sleep disorders, and the most common digestive symptoms, was used to inquire about the patient’s conditions. Symptoms were collected via a non-validated questionnaire based on patient self-reports. No standardized patient-reported outcome instruments (e.g., PSQI, EORTC, QLQ-STO22) were used, which may limit the comparability and granularity of symptom assessment. All included patients were being treated from January 2020 to June 2022 in this study. All patients were followed up at the first week after discharge (1W), the first month after discharge (1M), the third month after discharge (3M), and the sixth month after discharge (6M). Food intake, weight loss, sleep disorders, and the most common digestive symptoms were collected. The collected data were patients’ self-reports, and the most common complaints at the follow-up time. At the same time, patients were informed that chemotherapy-related symptoms should be ruled out. All these characteristics and clinical information were collected from our hospital information system. Based on the inclusion criteria and exclusion criteria, 578 patients were included in our study before PSM (**Figure 1**). A total of 149 patients were excluded because they either did not have gastric cancer (n=149), did not undergo radical gastrectomy (n=185), had insomnia before surgery (n=85), or had no completed data (n=102). After PSM, 144 patients were included in the DG group and 56 patients were included in the PG group when comparing the two groups. A total of 103 patients were included in the TG group, and 56 patients were included in the PG group when comparing the two groups. A total of 166 patients were included in the TG group, and 136 patients were included in the DG group when comparing the two groups. A total of 578 patients were enrolled in this study, and their baseline characteristics are summarized in **Table 1**. After propensity score matching, key baseline variables, including age, sex, and pathological stage, were well-balanced across all groups with no statistically significant differences (*P* > 0.05).

**Table 1:** Baseline clinicopathological characteristics before and after propensity score matching in the three groups.

		Before PSM			After PSM								
Variable	TG(n=193)	DG (n=325)	PG (n=60)	*P* value	DG(n=144)	PG(n=56)	*P* value	TG(n=103)	PG (n=56)	*P* value	TG(n=166)	DG(n=136)	*P* value
Sex													
Male	151	222	48	0.21	117	44	0.69	83	44	0.76	239	103	0.69
female	42	103	12		27	12		20	12		37	33	
**Age**	61.58±9.41	59.06±10.89	65.33±8.22	**0.00**	64.02±9.95	65.20±8.48	0.44	63.12±8.93	65.20±8.48	0.16	61.55±9.14	61.17±10.44	0.74
**Weight at discharge**	57.29±9.40	58.03±9.63	57.82±8.03	0.60	58.32±9.93	58.53±8.17	0.89	58.00±9.47	58.53±8.17	0.73	57.54±9.65	57.18±8.66	0.74
Pathological stage													
IA	24	87	14	**0.00**	38	13	0.97	22	13	0.70	22	22	0.92
IB	6	45	7		20	6		6	6		6	2	
IIA	22	26	8		15	7		16	7		22	18	
IIB	30	47	15		31	14		20	14		22	17	
IIIA	43	60	11		28	11		29	11		39	35	
IIIB	46	45	5		12	5		10	5		41	31	
IIIC	22	15	0		0	0		0	0		14	11	

Bold values indicate statistically significant differences (P < 0.05).

### Propensity score matching

Propensity score matching (PSM) was applied to decrease selection bias and compare follow-up data. Matching was utilized based on age, sex, and pathological stage. A 0.02 caliper width was used. When TG and DG were compared, a 1:1 matching ratio was performed. When TG and PG were compared, a 3:1 matching ratio was performed. When DG and PG were compared, a 3:1 matching ratio was performed. However, it should be noted that PSM was limited to age, sex, and pathological stage. Other clinically relevant factors such as tumor location, preoperative nutritional status, comorbidities, and reconstruction-specific variables were not included in the matching process, which may introduce residual confounding.

### Statistical analysis

For measurement data, Student’s t-test was used, and the mean difference (MD) with a 95% confidence interval (CI) to assess the outcomes. For enumeration data, the chi-squared (χ2) analysis or Fisher’s exact test was used, and the odds ratio (OR) with 95% CI to assess the outcomes. A value of *P* < 0.05 was considered statistically significant.

## Results

### Before PSM

#### Food intake

In most patients, food intake recovered to half of normal at 1W. There was no difference between the three surgical procedures. However, at 1M, food intake recovered more quickly in DG than in the other surgical procedures group (**Table 2**). 77% (218/325) of patients had recovered to normal 3/4 of their food intake in DG. Thus, the ratio was 52% (95/193) in TG and 55% (33/60) in the proximal group. Moreover, no significant differences were observed between groups at the third month and the sixth month.

**Table 2:** Food intake between the three groups before PSM.

Variable	TG (n=193)	DG (n=325)	PG (n=60)	*P* value
The first week after discharge				
Normal	0	1	0	1.00
3/4 normal	68	128	20	0.50
1/2 normal	119	188	38	0.57
1/4 normal	6	8	2	0.94
The first month after discharge				
Normal	8	13	1	0.70
3/4 normal	95	218	33	**0.00**
1/2 normal	76	90	23	**0.01**
1/4 normal	4	4	3	0.13
The third month after discharge				
Normal	163	267	52	0.61
3/4 normal	25	48	7	0.73
1/2 normal	4	10	1	0.69
1/4 normal	1	0	0	0.44
The sixth month after discharge				
Normal	160	272	53	0.98
3/4 normal	27	51	7	0.69
1/2 normal	6	2	0	0.07
1/4 normal	0	0	0	ref

Bold values indicate statistically significant differences (P < 0.05).

#### Sleep disorders

It amazed us that 46% of patients (263/578) experienced sleep disorders in 1W (**Table 3**). The patient reported with the most common sleep disorder symptom was easy to wake. Although this proportion had decreased, 20% of patients (123/578) also experienced sleep disorders at 1M. At 1M, the incidence of difficulty falling asleep was lower in DG than that in the other groups (*P* < 0.001). As time went on, this proportion further decreased. The incidence did not differ between the three groups at the third month and at 6M.

**Table 3:** Sleep disorders between the three groups before PSM.

Variable	TG (n=193)	DG (n=325)	PG (n=60)	*P* value
The first week after discharge				
Normal	104	181	30	0.71
Difficulty falling asleep	4	8	1	1.00
Easy to wake	85	136	29	0.62
The first month after discharge				
Normal	150	262	43	0.27
Difficulty falling asleep	11	1	1	**0.00**
Easy to wake	32	62	16	0.22
The third month after discharge				
Normal	187	312	59	0.60
Difficulty falling asleep	0	2	0	0.62
Easy to wake	6	11	1	0.84
The sixth month after discharge				
Normal	185	318	60	0.15
Difficulty falling asleep	2	5	0	1.00
Easy to wake	6	2	0	0.07

Bold values indicate statistically significant differences (P < 0.05).

#### Weight loss

**Figure 2:**
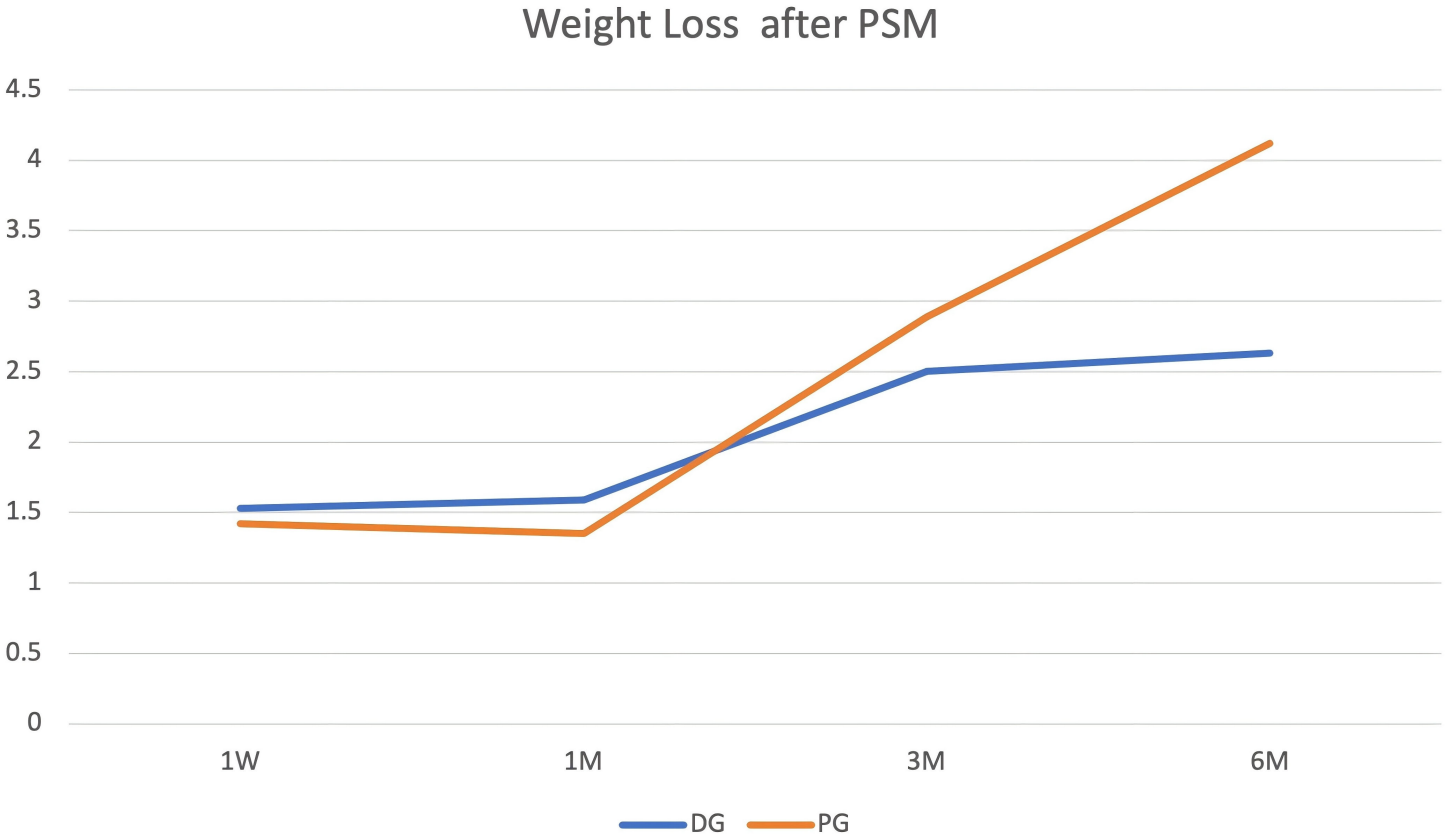
Body weight trajectories from baseline. The figure displays body weight measurements at baseline (defined as weight at discharge) and at subsequent time points during the follow-up period.

Weight at discharge was baseline during follow-up (**Figure 2**). In the three groups, weight loss did not differ at 1W (TG vs. DG vs. PG: 1.43 ± 1.72 vs. 1.47 ± 2.36 vs. 1.42 ± 1.24, *P* = 0.67) and at 1M (TG vs. DG vs. PG: 1.33 ± 2.27 vs. 1.37 ± 2.12 vs. 1.30 ± 1.55, *P* = 0.97). However, weight loss was lower in DG than the other groups at 3M (TG vs. DG vs. PG: 3.24 ± 3.39 vs. 2.33 ± 3. 14 vs. 2.87 ± 3.13, *P* = 0.01). As time went on, weight loss continued. However, weight loss was lowest in the DG at 6M (TG vs. DG vs. PG: 4. 14 ± 4.74 vs. 2.58 ± 4.11 vs. 4. 12 ± 4.55, *P* = 0.001).

#### Patient-reported main symptoms

The common complaints after gastrectomy were abdominal distention, abdominal pain, diarrhea, nausea and vomiting, and eating obstruction sensation (**Table 4**). No matter if its TG, DG, or PG, it was found that abdominal distention was the most common complaint, and abdominal pain was followed in this study. The incidence of eating obstruction sensation in DG was lower than the other two groups at 1W (*P* < 0.001) and at 1M (*P* < 0.001). These incidences of these symptoms were not significantly different between the three groups at 3M and at 6M.

**Table 4:** Main digestive symptoms between three groups before PSM.

Variable	TG (n=193)	DG (n=325)	PG (n=60)	*P* value
The first week after discharge				
No	65	132	21	0.26
Abdominal distension	78	119	20	0.53
Abdominal pain	31	57	11	0.88
Diarrhea	7	8	1	0.69
Nausea and vomiting	3	8	1	0.91
Eating obstruction sensation	9	1	6	**0.00**
The first month after discharge				
No	93	170	31	0.67
Abdominal distension	54	92	15	0.87
Abdominal pain	26	41	8	0.80
Diarrhea	7	10	0	0.34
Nausea and vomiting	2	9	1	0.43
Eating obstruction sensation	11	3	5	**0.00**
The third month after discharge				
No	183	310	58	0.84
Abdominal distension	1	5	0	0.58
Abdominal pain	0	0	0	ref
Diarrhea	3	1	0	0.32
Nausea and vomiting	5	8	1	1.00
Eating obstruction sensation	1	1	1	0.32
The sixth month after discharge				
No	186	317	59	0.70
Abdominal distension	1	1	0	1.00
Abdominal pain	1	1	0	1.00
Diarrhea	1	3	0	1.00
Nausea and vomiting	3	2	0	0.51
Eating obstruction sensation	1	1	1	0.32

Bold values indicate statistically significant differences (P < 0.05).

### After PSM

#### Food intake

Food intake recovered to half of the normal diet at 1W in most patients. Compared with the TG group and DG group, food intake recovered more quickly in PG at 1W (*P* < 0.05). Food intake of three patients recovered to a normal diet in the PG at 1W. However, no patient recovered to a normal diet in TG and DG (**Table 5**). At 3M, 66% of patients (90/136) had recovered to a 3/4 normal diet in DG, and 54% of patients (89/166) had recovered to a 3/4 normal diet in TG. There was a significant difference between the two groups (*P* = 0.03). For most patients, food intake recovered to the normal diet at 3M. There were no significant differences between the three groups.

**Table 5:** Food intake between the three groups after PSM.

	DG vs PG			TG vs PG			TG vs DG		
Variable	DG (n=144)	PG (n=56)	*P* value	TG (n=103)	PG (n=56)	*P* value	TG (n=166)	DG (n=136)	*P* value
The first week after discharge									
Normal	0	3	**0.02**	0	3	**0.04**	1	1	1.00
3/4 normal	55	20	0.87	34	20	0.86	62	63	0.13
1/2 normal	86	31	0.63	68	31	0.23	101	71	0.16
1/4 normal	3	2	0.62	1	2	0.55	2	1	1.00
The first month after discharge									
Normal	8	2	0.43	5	2	1.00	13	5	0.15
3/4 normal	92	28	0.08	58	28	0.51	89	90	**0.03**
1/2 normal	41	23	0.09	39	23	0.74	62	39	0.14
1/4 normal	3	3	0.35	1	3	0.13	2	2	1.00
The third month after discharge									
Normal	115	48	0.42	83	48	0.52	136	111	1.00
3/4 normal	24	7	0.52	18	7	0.50	25	22	0.87
1/2 normal	5	1	1.00	1	1	1.00	4	3	1.00
1/4 normal	0	0	ref	1	0	1.00	1	0	1.00
The sixth month after discharge									
Normal	115	49	0.23	82	49	0.28	133	110	0.89
3/4 normal	28	7	0.30	18	7	0.50	27	26	0.55
1/2 normal	1	0	1.00	3	0	0.55	4	0	0.09
1/4 normal	0	0	ref	0	0	ref	0	0	ref

Bold values indicate statistically significant differences (P < 0.05).

#### Sleep disorders

More than 40% of patients underwent sleep disorders (**Table 6**). Easy to wake and difficulty falling asleep were the complaints from our patients. No matter if it is TG, DG, or PG, easy to wake was the main complaint from those with the insomnia symptom. As time went on, this proportion further decreased. The incidence did not differ between the three groups at the first week, the first month, the third month, and 6M (*P*>0.05).

**Table 6:** Sleep disorders between the three groups after PSM.

	DG vs PG			TG vs PG			TG vs PG		
Variable	DG (n=144)	PG (n=56)	*P* value	TG (n=103)	PG (n=56)	*P* value	TG (n=166)	DG (n=136)	*P* value
The first week after discharge									
Normal	80	29	0.64	57	29	0.74	92	83	0.35
Difficulty falling asleep	7	2	1.00	3	2	1.00	4	1	0.37
Easy to wake	57	25	0.51	43	25	0.74	70	52	0.56
The first month after discharge									
Normal	117	39	0.13	86	39	0.05	139	110	0.55
Difficulty falling asleep	2	0	1.00	0	1	1.00	1	0	1.00
Easy to wake	25	17	0.05	17	16	0.10	26	26	0.26
The third month after discharge									
Normal	133	55	0.19	99	55	0.66	160	133	0.52
Difficulty falling asleep	1	0	1.00	0	0	ref	0	2	0.20
Easy to wake	10	1	0.30	4	1	1.00	6	1	0.13
The sixth month after discharge									
Normal	144	56	ref	144	56	ref	166	136	ref
Difficulty falling asleep	0	0	ref	0	0	ref	0	0	ref
Easy to wake	0	0	ref	0	0	ref	0	0	ref

#### Weight loss

**Figure 3:**
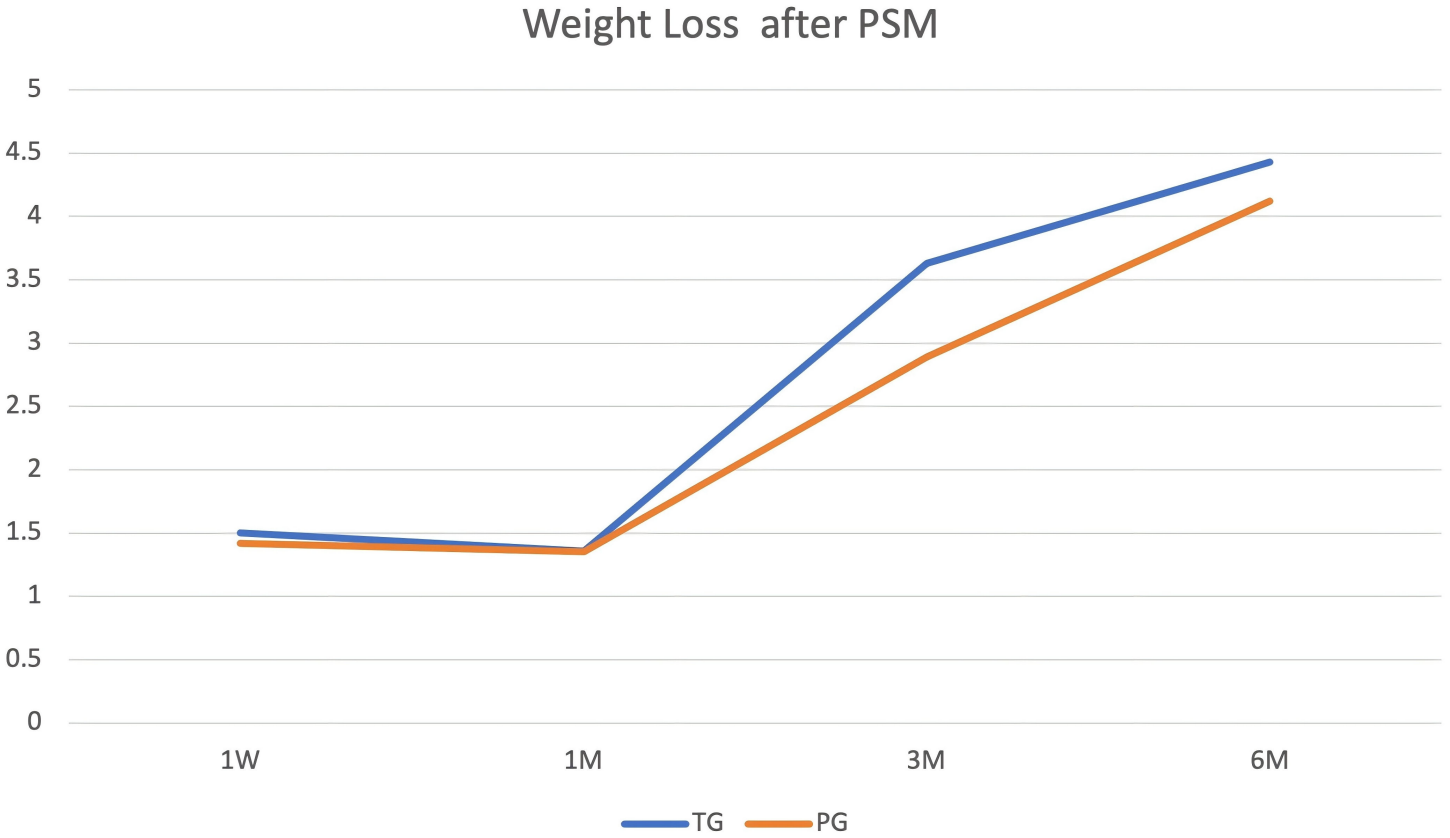
Weight loss outcomes. Body weight reduction was sustained through the 6-month (6M) follow-up period, with differences observed among the three study groups.

**Figure 4:**
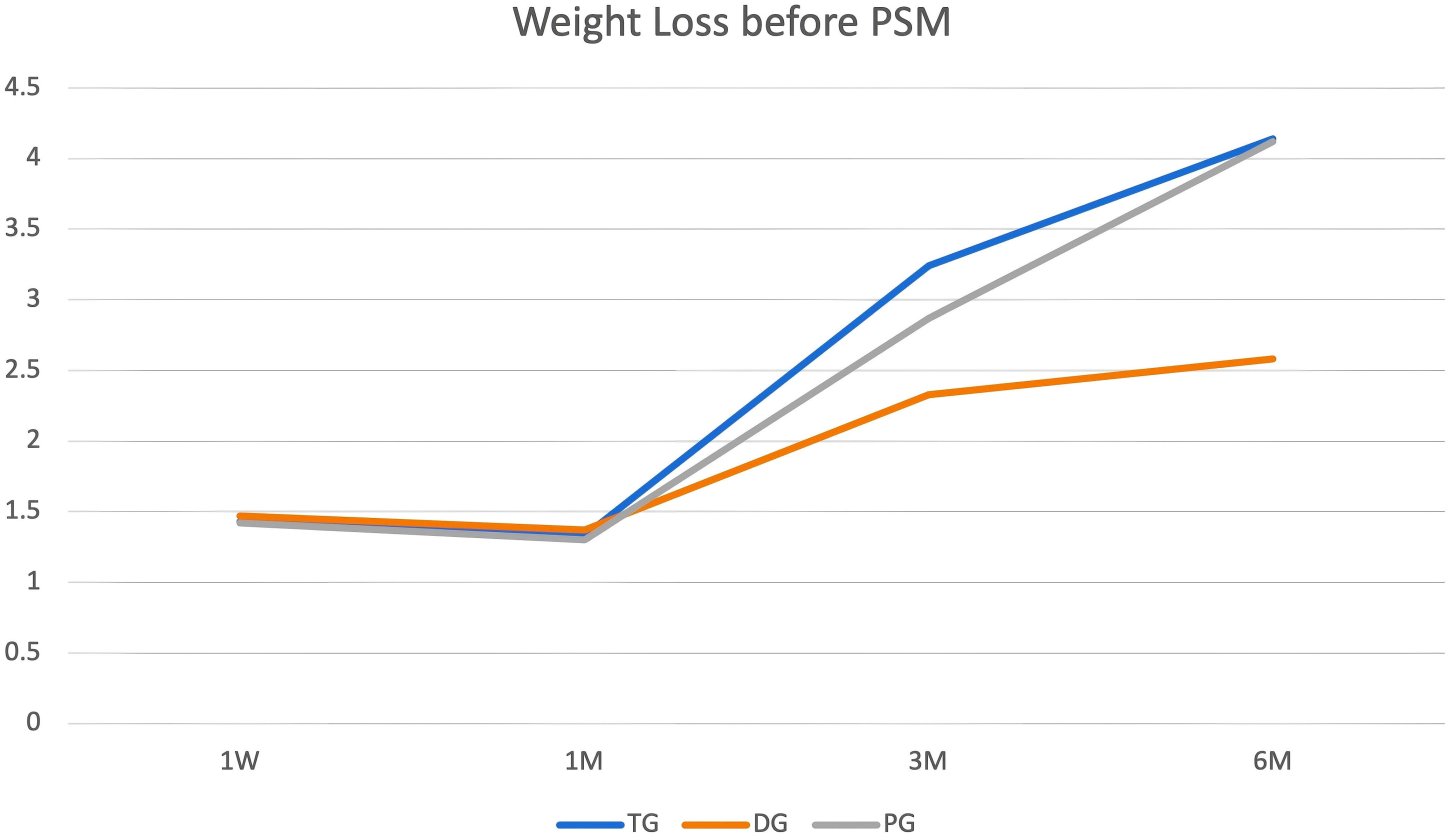
Weight loss outcomes. Body weight reduction was sustained through the 6-month (6M) follow-up period, with differences observed among the three study groups.

**Figure 5:**
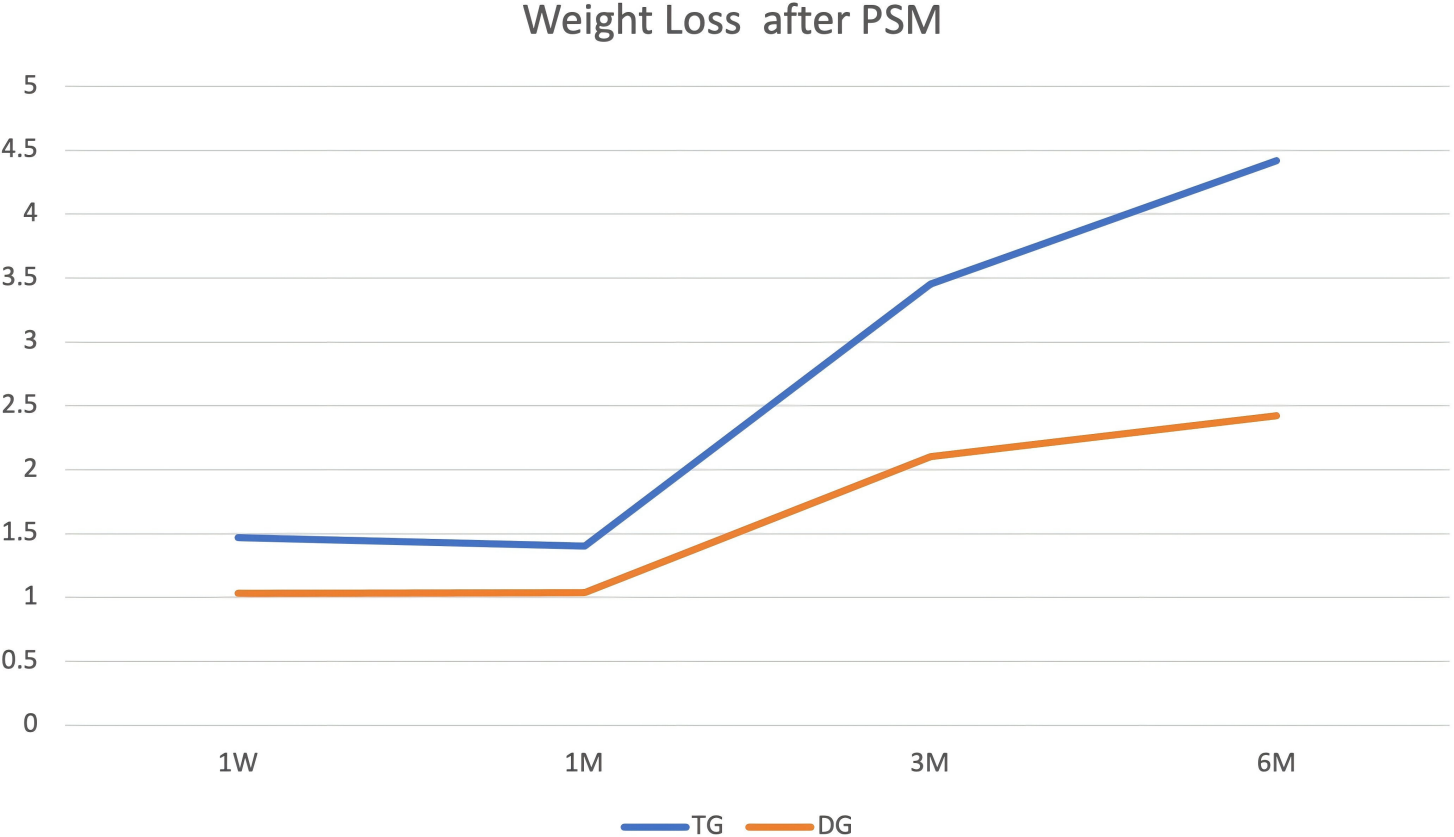
Weight loss outcomes. Body weight reduction was sustained through the 6-month (6M) follow-up period, with differences observed among the three study groups.

In our follow-up time, weight loss sustained for 6M. The differences among the three groups are shown in **Figures 3**, **4**, **5**. Compared with TG, weight loss was lower in DG at 1W (DG vs. PG: 1.53 ± 2.04 vs. 1.42 ± 1.28, *P* = 0.69, TG vs. PG: 1.50 ± 1.86 vs. 1.42 ± 1.28, *P* = 0.78, TG vs. DG: 1.47 ± 1.74 vs. 1.03 ± 1.56, *P* = 0.02). At 1M, weight loss was not different among the three groups (DG vs. PG: 1.59 ± 2.33 vs. 1.35 ± 1.58, *P* = 0.48, TG vs. PG: 1.36 ± 2.15 vs. 1.35 ± 1.58, *P* = 0.97, TG vs. DG: 1.40 ± 2.20 vs. 1.04 ± 1.84, *P* = 0. 12). However, weight loss was lower in DG than that in TG at 3M (DG vs. PG: 2.50 ± 2.97 vs. 2.89 ± 3.21, *P* = 0.42, TG vs. PG: 3.63 ± 3.15 vs. 2.89 ± 3.21, *P* = 0.16, TG vs. DG:3.45 ± 3.44 vs. 2. 10 ± 3.19, *P* < 0.001). At 6M, weight loss continued. Compared with PG and TG, weight loss was lowest in DG (DG vs. PG: 2.63 ± 3.80 vs. 4. 12 ± 4.59, *P* = 0.02, TG vs. PG: 4.43 ± 4.46 vs. 4. 12 ± 4.59, *P* = 0.68, TG vs. DG: 4.42 ± 4.74 vs. 2.42 ± 4.20,*P* < 0.001). Although weight loss was lower in PG than in TG, the difference was not statistically significant (*P*>0.05).

#### Patient-reported main symptoms

After matching, it was also found that abdominal distention was the most common complaint after gastrectomy (**Table 7**). The second common patient-reported symptom was abdominal pain. At 1W, incidences of main symptoms were significantly different between the three groups. Compared with DG, the incidence of main symptoms was higher in PG (88/144 vs. 36/56, *P* = 0.01). Compared with PG, the incidence of main symptoms was higher in TG (111/166 vs. 70/136, *P* = 0.01). Compared with DG, the incidence of eating obstruction sensation was higher in PG (6/56 vs. 1/144, *P* < 0.001). and in TG (9/166 vs. 1/136, *P* = 0.03). However, compared with TG, the incidence of abdominal pain was higher in PG (11/56 vs. 12/103, *P* = 0.03). At 1M, incidences of most of symptoms in PG decreased. Compared with TG, the incidence of digestive symptoms was lower in PG (87/166 vs. 53/136, *P* = 0.02). It was found that the incidence of eating obstruction sensation was lower in DG than that in PG (5/56 vs1/144, *P* = 0.01) and that in TG (11/166 vs. 2/144, *P* = 0.04). These symptoms typically subside over time. In our experience, oral non-steroidal anti-inflammatory drugs (NSAIDs) and antispasmodics are viable options for pain management and have demonstrated efficacy in alleviating pain. At the third and sixth months after discharge, most patients had no obvious digestive discomfort.

**Table 7:** Main symptoms between three groups after PSM.

	DG vs PG			TG vs PG			TG vs PG		
Variable	DG (n=144)	PG (n=56)	*P* value	TG (n=103)	PG (n=56)	*P* value	TG (n=166)	DG (n=136)	*P* value
The first week after discharge									
Normal	63	20	**0.01**	32	20	0.51	55	66	**0.01**
Abdominal distension	51	17	0.51	44	17	0.17	69	45	0.15
Abdominal pain	21	11	0.40	12	11	**0.03**	25	19	0.87
diarrhea	3	1	1.0	6	1	0.68	7	4	0.76
Nausea and vomiting	5	1	1.0	1	1	0.48	1	1	1.0
Eating obstruction sensation	1	6	**0.00**	8	6	0.22	9	1	**0.03**
The first month after discharge									
Normal	90	30	0.26	49	30	0.51	79	83	**0.02**
Abdominal distension	31	12	1.00	26	12	0.70	44	33	0.69
Abdominal pain	14	8	0.45	12	8	0.80	23	14	0.38
diarrhea	3	0	0.56	6	0	0.10	7	2	0.19
Nausea and vomiting	5	1	1.0	1	1	1.00	2	2	1.00
Eating obstruction sensation	1	5	**0.01**	9	5	1.00	11	2	**0.04**
The third month after discharge									
Normal	131	54	0.24	98	54	1.00	156	131	0.43
Abdominal distension	8	0	0.11	0	0	ref	1	2	0.59
Abdominal pain	0	0	ref	0	0	ref	0	0	ref
diarrhea	1	0	1.00	2	0	0.54	3	0	0.26
Nausea and vomiting	3	1	1.00	2	1	1.00	5	3	0.73
Eating obstruction sensation	1	1	0.48	1	1	1.00	1	0	1.00
The sixth month after discharge									
Normal	140	56	0.58	101	56	0.54	159	133	0.52
Abdominal distension	0	0	ref	1	0	1.00	1	1	1.00
Abdominal pain	0	0	ref	0	0	ref	1	0	1.00
diarrhea	2	0	1.00	0	0	ref	1	2	0.59
Nausea and vomiting	2	0	1.00	1	0	1.00	3	0	0.26
Eating obstruction sensation	0	0	ref	0	0	ref	1	0	1.00

Bold values indicate statistically significant differences (P < 0.05).

## Discussion

Radical gastrectomy has been performed to prolong the overall survival of GC patients (28, 29). However, curative resection is not only the cornerstone of treatment for this disease, but also the key to improving quality of life (30, 31). In surgical studies, improved operation techniques were used to better clinical digestive symptoms (32–34). In fact, gastrectomy itself could lead to digestive discomfort. However, existing research findings found that the impact of the scope of gastrectomy on quality of life was controversial (7, 35). These discomforts seem to be difficult to reverse and reduce the quality of life in the short term. So, patient-reported discomforts after gastrectomy should be given an increasing amount of attention (36). In our studies, patient-reported main discomforts were collected and compared among TG, DG, and PG. Our results showed that food intake could recover to half of normal in most patients at 1W, three-fourths of normal in the first month. This patient-reported symptom did not differ among the three groups. A prospective observational study showed that food intake loss was greater in TG than in DG (37). In our study, food intake loss was similar among TG, DG, and PG. It told us that regardless of the size of scope of gastrectomy, it had an impact on food intake. So, it is important to guide the patient to increase the frequency of food intake. We found that approximately 40% of patients had sleep disorders, no matter TG, DG, and PG. By the third month after gastrectomy, sleep disorders had improved. Cancer patients always experience varying degrees of anxiety. Anxiety could lead to sleep disorders (38). A meta-analysis showed that sleep disorders were a risk factor for chronic postsurgical pain (39). Meanwhile, a limited number of studies have suggested that sleep disturbances and anxiety states may be associated with enhanced immune function and reduced inflammatory markers (40). Moreover, sleep disorders also have an impact on metabolism and nutrition (41). Sleep disorders may play an important role in postoperative malnutrition in gastric cancer patients. So, improving sleep could enhance the nutritional status of patients who have undergone gastrectomy. PG with double-tract anastomosis was widely considered as a function-preserving operation due to it could offer an advantage in postoperative nutritional parameters (42, 43). However, in our follow-up, weight loss was maximum in TG and was minimum in DG. This result suggested that preservation of the cardia was important to weight maintenance and indicated that function-preserving surgery is very important to improve quality of life for gastric cancer patients. In our results, abdominal distention, abdominal pain, diarrhea, nausea and vomiting, and eating obstruction sensation were the main patient self-reports ‘ digestive symptoms. In these symptoms, the incidence of abdominal distention was highest, no matter if it is TG, DG, or PG. Digestive function decreased in most patients after gastrectomy (44, 45). Abdominal distention was one of the manifestations of decreased digestive function. Moreover, eating obstruction was more frequent in TG and PG than in DG. Esophagojejunal anastomosis could lead to anastomosis strictures and reflux esophagitis (46–48). It had an impact on eating obstruction due to a lack of cardia. However, our study also has some limitations. Firstly, as a retrospective single-center study, this research cannot avoid the inherent potential biases associated with this type of design. Although we used PSM to balance known confounding factors such as age, sex, and pathological stage, we could not control for unmeasured or unavailable variables, including tumor location, baseline nutritional status, and comorbidities, which are known to influence both surgical selection and postoperative outcomes. For instance, the decision to perform a specific procedure (total, distal, or proximal gastrectomy) was heavily influenced by factors such as the precise location of the tumor, preoperative nutritional status, and patient comorbidities—all of which could also impact postoperative recovery and quality of life. In particular, more advanced tumor stages or poorer baseline conditions themselves might be associated with more severe postoperative symptoms, potentially leading to biased estimation of the effect of the surgical procedure itself. Secondly, patient-reported symptoms were not collected from scales, may have self-report biases, and were not quantitatively analyzed. Moreover, patient-reported symptoms were not collected using validated or standardized PRO instruments, which may introduce recall bias and limit the quantitative analysis of symptom severity and impact. Furthermore, extensive pairwise comparisons were conducted across three surgical groups, multiple symptoms, and four timepoints without adjustment for multiple testing. This increases the risk of Type I error, and thus the reported differences should be interpreted with caution. A prospective study is needed to further validate these conclusions. Therefore, future research should prioritize prospective, multicenter studies with long-term follow-up to enroll a broader patient population. It is essential to develop more comprehensive propensity score matching (PSM) or statistical models that incorporate variables potentially influencing both surgical selection and postoperative outcomes. These may include regional characteristics of the patient cohort, tumor invasiveness and biological behavior, the presence of comorbidities, and specific surgical details. Additionally, the follow-up process should integrate widely recognized assessment tools such as the Pittsburgh Sleep Quality Index (PSQI) and gastrectomy-specific patient-reported outcome (PRO) instruments, for example, the EORTC QLQ-STO22 or PGSAS-45.

## Conclusions

Compared with TG and PG, DG was associated with significantly less weight loss and a lower incidence of digestive symptoms during the six-month follow-up period. These findings suggest that DG may better preserve digestive and nutritional function, likely due to the retention of a larger gastric reservoir and more physiological continuity of the gastrointestinal tract. The preservation of the cardia in DG may also contribute to reduced reflux and eating obstruction, thereby improving postoperative quality of life. This study is to highlight that sleep disorders and abdominal distension are among the most frequently reported complaints following gastrectomy, regardless of the surgical approach. These symptoms should be actively addressed in postoperative care to enhance recovery and quality of life. Specifically, clinicians should consider incorporating routine assessment and management of sleep quality and abdominal comfort into follow-up protocols. Nutritional counseling, dietary modifications, and potential pharmacological or behavioral interventions for sleep improvement may be beneficial. Future research should focus on developing standardized patient-reported outcome measures for gastrectomy-specific symptoms and exploring targeted interventions to alleviate sleep disturbances and abdominal distension. In clinical practice, these findings support the consideration of DG when oncologically feasible and emphasize the need for personalized postoperative support tailored to the type of gastrectomy performed.

## Data Availability

The original contributions presented in the study are included in the article/supplementary material. Further inquiries can be directed to the corresponding author.
